# Lymphoma-Associated Multifocal Motor Neuropathy With Conduction Block in a Patient Recently Diagnosed With HIV

**DOI:** 10.7759/cureus.87568

**Published:** 2025-07-08

**Authors:** Philip B Adebayo, Mohammed Mukasa, Doreen Mathew, Nuru Saleh, Caroline Ngimba

**Affiliations:** 1 Neurology, Aga Khan University, Dar es Salaam, TZA; 2 Internal Medicine, Aga Akhan University, Dar es Salaam, TZA; 3 Oncology, Aga Khan Health Service, Dar es Salaam, TZA; 4 Pathology, Aga Khan Health Service, Dar es Salaam, TZA

**Keywords:** conduction block, demyelination, hiv, multifocal motor neuropathy, non-hodgkin’s lymphoma

## Abstract

Multifocal motor neuropathy with conduction block (MMNCB) is a rare acquired immune-mediated demyelinating neuropathy that affects peripheral motor nerves, presenting as asymmetric, usually upper limb weakness without sensory impairment. Its concurrence with non-Hodgkin lymphoma (NHL) is an exceedingly rare phenomenon. This case describes a patient who presented with sequential bilateral foot drop starting from the left foot, with an ensuing weak hand grip. She was discovered to have cervical and occipital lymphadenopathy, which occasioned further blood work and tissue diagnosis. Her electrodiagnostic studies showed multiple asymmetric motor conduction blocks, normal distal motor latencies, and sensory studies consistent with a diagnosis of MMNCB. Her lymph node biopsy revealed diffuse large B-cell lymphoma (DLBCL). She could not afford intravenous immunoglobulin (IVIG); hence, pulse intravenous methylprednisolone was tried with no response. While her blood work showed a new diagnosis of HIV infection, her antiganglioside antibody panel was negative for anti-GM1 antibodies. Fortunately, her weakness began to improve in the third month of her chemotherapy with rituximab, cyclophosphamide, doxorubicin, vincristine, and prednisolone (R-CHOP). Case reports have only provided the association of MMNCB and lymphoma. How each condition influences the other's manifestation, treatment, and prognosis is still poorly understood.

## Introduction

First reported in the 1980s by Roth et al. [1], multifocal motor neuropathy with conduction block (MMNCB), also referred to as multifocal motor neuropathy (MMN) [2], is still a rare peripheral nerve disorder with a reported prevalence of less than two per 100,000 [3, 4].

It is an acquired immune-mediated demyelinating neuropathy characterized by slowly progressive, predominantly distal, asymmetric limb weakness affecting mostly upper extremities in comparison to lower extremities, with minimal or no sensory involvement, and by the presence of persistent multifocal conduction blocks of the affected motor nerves in the non-compressed areas [4]. Despite decades of research, the exact etiopathogenesis of MNN is still unclear [2]. Nevertheless, it tends to affect more males than females, with a ratio of about 3:1, with the average age at presentation of 40 years (range: 30-50 years) [5, 6].

Neurological symptoms in solid malignancies have multiple etiopathogeneses, which could include metastasis or direct infiltration by the tumor and, in some instances, known indirect mechanisms such as toxicity, ectopic secretion of hormones, or induced coagulopathies (paraneoplastic neurological syndromes). The occurrence of immune-mediated peripheral nerve demyelination, such as MMN, in the setting of solid malignancies, is rare, with a prevalence of less than 1% reported in some studies [7]. Rarer still is incident MMNCB in lymphomas compared to other solid malignancies like lung, breast, and ovarian cancers [8]. Although a clear linkage has not been fully elucidated, some investigators did demonstrate a monoclonal IgM anti-GM1 autoantibody produced by B-cell lymphoma using immunofluorescent flow cytometric analysis of lymphoma cells CD19 versus various immunoglobulin chains or CD79b. The antibody was shown to bind to the Galβ1-3GalNAc terminal disaccharide residues of glycolipids GM1 and GD1b, which are abundant in peripheral nerve myelin [9].

In non-Hodgkin lymphomas (NHL), the prevalence of peripheral neuropathies, whether paraneoplastic or otherwise, is approximately 5%. Concerning MMN in particular, its coexistence or association with NHL is much more uncommon [10,11] and has only been reported in two case reports [9,12]. We report the rare case of a middle-aged female patient who presented to the neurology clinic with sequential foot drop and cervical lymphadenopathy. We emphasize the importance of early electrodiagnostic testing, among other pertinent investigations, for prompt diagnosis.

## Case presentation

A 42-year-old female patient presented to our outpatient neurology clinic with insidiously progressive weakness of the left foot for about one month. She reported neither pain nor abnormal sensation in the left lower limb or other body parts. She had no weakness in any other limbs. There was no history of preceding trauma, back pain, leg swelling, or any other constitutional symptoms. She has no underlying medical illness. She neither smoked nor drank alcoholic beverages. She had an enlarged, rubbery, non-tender left anterior cervical lymph node measuring 2.0 x 2.5 cm. Other general examination findings were unremarkable. Her neurological examination revealed weakness of the dorsiflexors of the left foot (Medical Research Council (MRC) scale of 2/5). There was no weakness in any other groups of muscles. She had no objective sensory deficit. Her ankle jerk was absent on the left and depressed on the right. Other muscle stretch reflexes were normal. The patient was suspected of having a left peroneal neuropathy to exclude an L4/L5 radiculopathy. She was reluctant to pursue any extensive investigation because she felt her only issue was her left foot drop. She also lacked comprehensive health insurance coverage.

Following her initial hesitations and subsequent convincing, her blood work, magnetic resonance imaging (MRI) of the brain, spine, and abdomen, nerve conduction test and electromyogram, and lymph node biopsy were eventually arranged. Table 1 shows the results of the blood work and cerebrospinal fluid (CSF) analysis.

**Table 1:** Summary of the patient’s hematological, chemistry, and CSF work-up CSF: cerebrospinal fluid; CRP: C-reactive protein; BUN: blood urea nitrogen; ALT: alanine transaminase; AST: aspartate transaminase; ESR: erythrocyte sedimentation rate; AFB: acid-fast bacilii  CD4: cluster of differentiation 4

Laboratory test	Value	Reference range
White blood count x10^9^/L	6.45	4-11
Hemoglobin, g/dL	10	12.5-16.5
Platelets counts, x 10^9^/L	274	150-450
Creatinine, umol/L	46	60-110
Sodium, mmol/L	135	135-145
Potassium, mmol/L	3.4	3.5-5
BUN	4.8	1.79 - 6.43
Total bilirubin	15	0 - 21
ALT, umol/L	22	10-40
AST, umol/L	25	10-40
Uric acid, umol/L	291.5	142-339
Vitamin B12, pg/ml	547.3	191-663
ESR	26	0 - 20
CRP, mg/dL	6	0.5-5
CSF glucose	2.43	
CSF protein	49.60	15 - 45
CSF AFB	Negative	Negative
CSF ganglioside antibodies; (GM1, GM2, GM3, GD1a, GD1b, GT1b, GQ1b )	Negative	Negative
HIV serology	Positive	Negative
CD4, cells/mm^3^	504	>500

Nevertheless, her weakness continued to worsen, and by the third month after the initial visit, she had developed a right foot drop similar to the left foot weakness, while the left foot drop had worsened. At this point, she needed bilateral ankle support. In addition to her weakness, she reported intermittent muscle cramps in both thighs. She also noted a slight weakness of her right-hand grip, evidenced by a minor difficulty opening jars and a mild loss of finger dexterity. She had no new sensory symptoms or bladder or bowel dysfunction. She had denied any new constitutional symptoms. Her repeat neurological examination revealed a bilateral high-steppage gait. She had no atrophy, fasciculation, or abnormal movements. Her muscle tone and bulk were normal and symmetrical. However, muscle weakness was more predominant in the distal compared to the proximal lower limb muscles, with a power of 1/5 (MRC) in the left foot plantar flexors and dorsiflexors and a power of 2/5 (MRC) in the right foot plantar flexors and right foot dorsiflexors. Power in the hip adductors/abductors and the hip flexors was 3+/5 (MRC) on the left compared to 4/5 (MRC) in the corresponding muscle groups on the right. Knee and ankle jerk reflexes were bilaterally absent, and the plantar response was flexor bilaterally. She had no new systemic examination findings.

**Figure 1:**
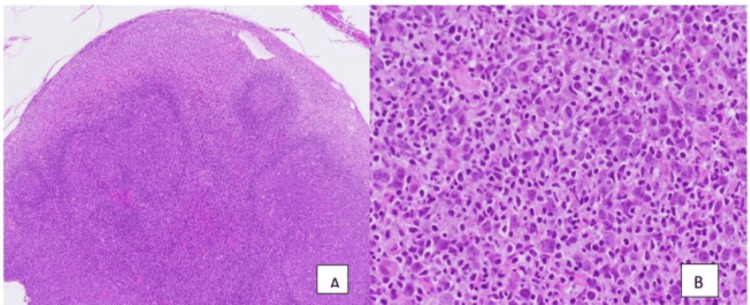
Lymph node histology: hematoxylin and eosin sections A, low power: The lymph node architecture is mostly preserved with scattered, enlarged, reactive-appearing follicles with active, polarized germinal centers and intact mantle zones. Within the subcapsular sinus, there is marked distention by an atypical infiltrate of large lymphoid cells with round nuclei, dispersed chromatin, multiple prominent nucleoli, and moderately abundant eosinophilic cytoplasm. B, high power: The atypical lymphoid infiltrate is associated with frequent small lymphocytes, neutrophils, and apoptotic debris. Scattered mitotic figures are seen.

Lymph node biopsy for histology and immunohistochemistry confirmed a diagnosis of diffuse large B-cell lymphoma (DLBCL) with lymphoplasmacytic/lymphoblastic differentiation (Figure 1).

**Figure 2:**
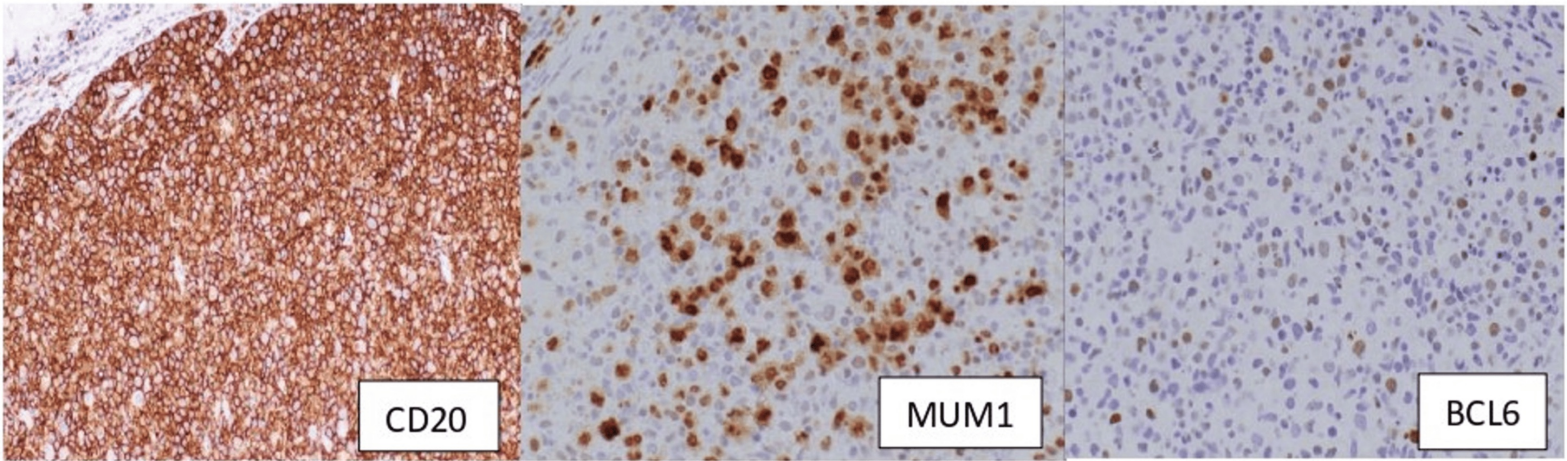
The patient's immunohistochemistry report Sinusoidal large atypical cells are strongly and diffusely positive for CD20, consistent with B cells, with co-expression of MUM1 and BCL6 (weak).

There was focal lambda light chain restriction within the neoplastic cells in the immunohistochemistry (Figure 2) for kappa and lambda immunoglobulin light chains. The large B cells were negative for CD30, ALK1, and human herpesvirus-8 (HHV-8) by immunohistochemistry and Epstein-Barr virus (EBV) by Epstein-Barr virus-encoded small RNA (EBER) in situ hybridization. An immunohistochemical stain for cytomegalovirus (CMV) was negative. The background small lymphocytes were mostly CD20+ B cells and fewer CD3+ T cells. Reactive follicles were positive for CD20, with the germinal center subset positive for BCL6, and were encompassed by CD21+ follicular dendritic cell meshwork. Numerous CD3+ T cells were present in the interfollicular areas.

**Figure 3:**
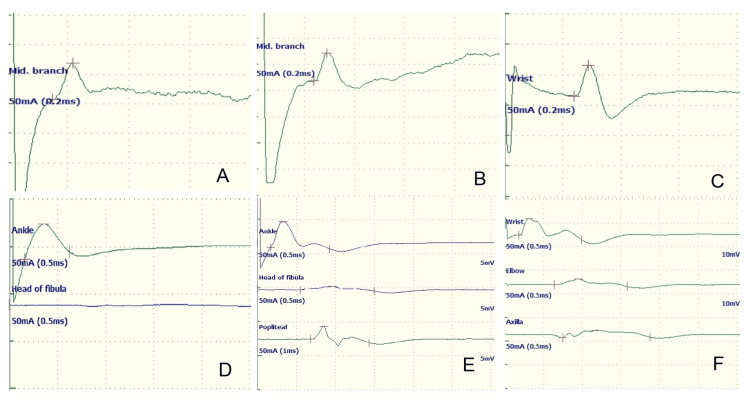
Sample images of compound muscle action potentials and sensory action potentials A-C: Normal superficial peroneal (right), superficial peroneal (left), and median (right) sensory nerve action potentials, respectively; D-F: Conduction block and temporal dispersion noted in the left common peroneal, right common peroneal, and right median compound muscle action potential, respectively.

Her motor conduction study revealed a definite conduction block across the fibula neck of both peroneal nerve compound muscle action potentials (CMAP). Median nerve CMAP showed a conduction block in the forearm, ulnar nerve CMAP showed a conduction block across the elbow, and a probable conduction block of the left tibial nerve CMAP across the popliteal fossa was noted. All sensory conduction studies were normal (Figure 3).

**Figure 4:**
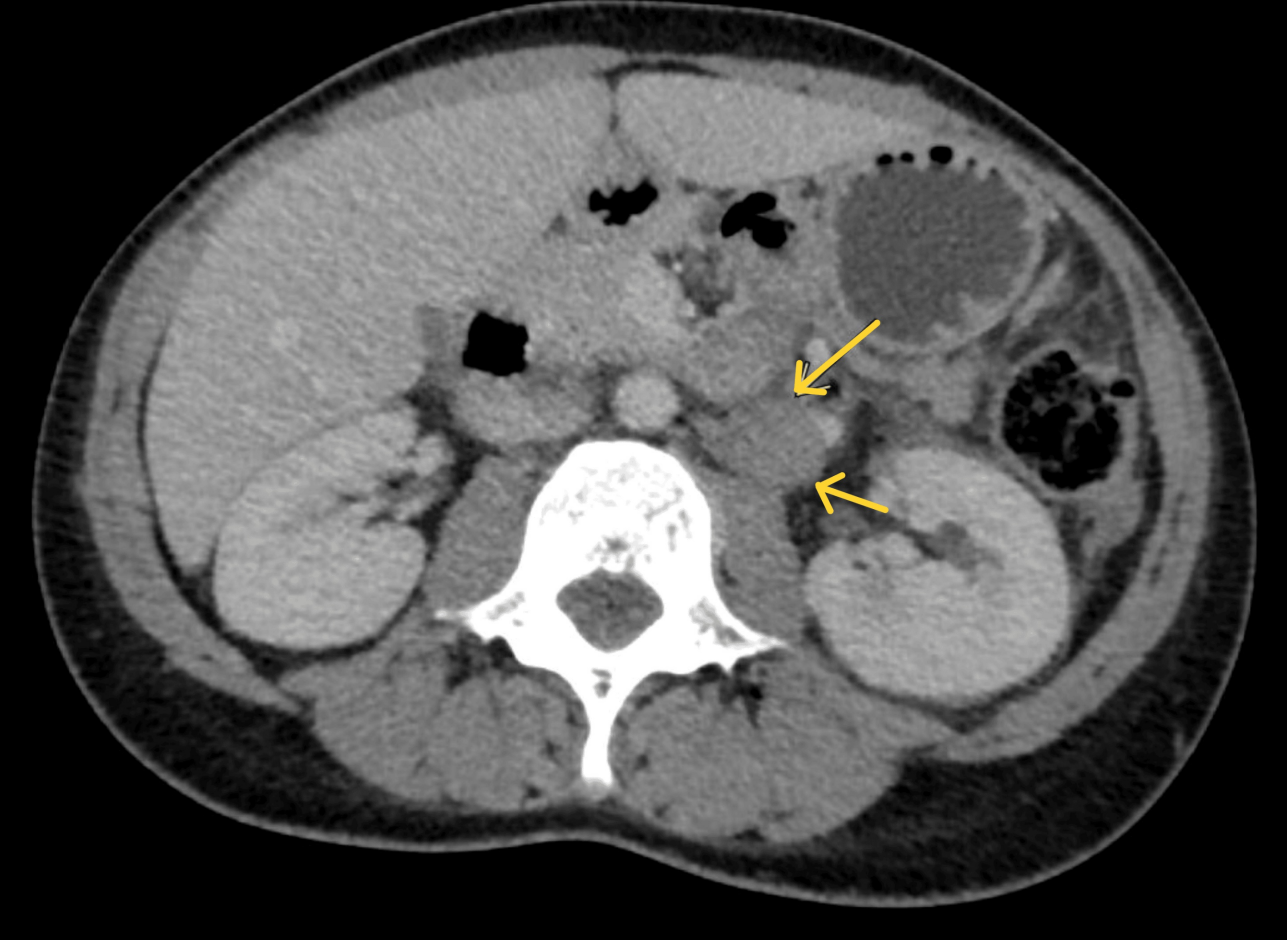
Axial computerized tomography (CT) scan of the abdomen The arrows indicate left paraaortic lymph node enlargement.

**Figure 5:**
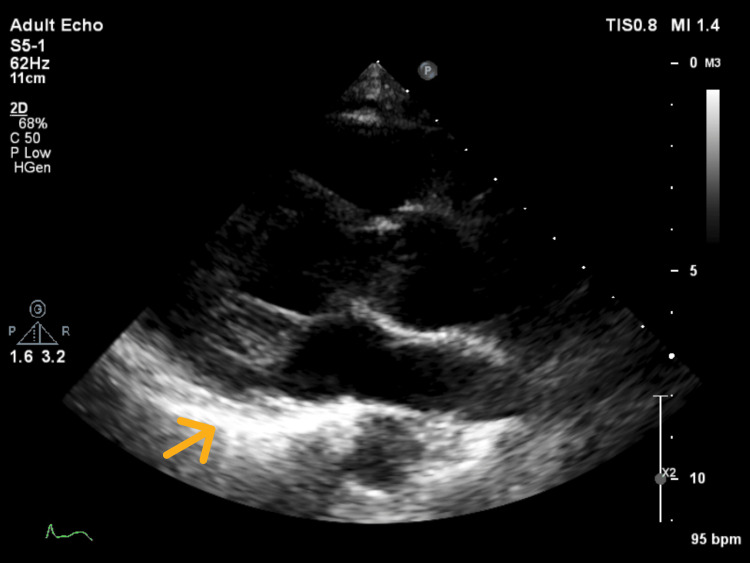
Two-dimensional echocardiogram The arrow indicates a thickened and shinning pericardium.

Computed tomography (CT) for metastatic workup revealed an enlarged left paraaortic node measuring 1.9 x 2.9 cm (Figure 4) and another mesenteric node at the left infrarenal region measuring 3.3 x 3.2 cm. There were no significant inguinal nodes. The liver was also enlarged (18 cm) and homogenous in attenuation with no focal parenchymal lesions. MRI of the brain and whole spine did not reveal any intracranial lesion or tumor infiltration of the spinal cord, the radicles, or the plexuses. The echocardiogram revealed concentric remodeling, diastolic dysfunction with preserved ventricular systolic functions, and a thickened and shining pericardium with constrictive physiology consistent with constrictive pericarditis (Figure 5).

The patient was tried on five days of intravenous methylprednisolone when it was apparent she could not afford intravenous immunoglobulin (IVIG). She did not respond to this therapy. She started daily antiretroviral treatment (ART) consisting of tenofovir disoproxil fumarate (300 mg), lamivudine (300 mg), and dolutegravir (50 mg). Four weeks into the ART therapy, and with no neurological improvement, she commenced six cycles of an R-CHOP (short for rituximab, cyclophosphamide, doxorubicin, vincristine, and prednisolone) chemotherapy regimen, with each cycle spaced three weeks (21 days) apart. The treatment included IV rituximab 500 mg on Day 1, IV cyclophosphamide 1100 mg on Day 1, IV doxorubicin 70 mg on Day 1, and IV vincristine 2 mg on Day 1. In addition, she used prednisone 100 mg orally from Days 1 to 5 as per the National Comprehensive Cancer Network (NCCN) guidelines for NHL, mainly DLBCL [13]. This protocol combines both immunotherapy and chemotherapy to target malignant B cells effectively. Her neurological review after the third circle revealed a power improvement of MRC 5/5 of the right wrist and forearm flexors, 3/5 of the foot dorsiflexors bilaterally, MRC 4+/5 of the right plantar flexors, and MRC 4/5 of the left plantar flexors. She had stopped using her foot support before the clinic visit.

## Discussion

Although MMN was discovered over 30 years ago, it remains a rare phenomenon. The criteria for its definitive diagnosis have been agreed upon. It includes three clinical and electro-diagnostic criteria [14] as follows: motor weakness (in the absence of a sensory loss save for a slight vibration impairment) in the distribution of two or more nerves for more than a month, presence of a definite motor conduction block in at least one motor nerve with intact sensory nerve conduction, and absence of exclusion criteria, which include upper motor nerve lesion, early symmetric weakness of the involved limbs early in the course of the illness, significant sensory involvement (except the minor loss of vibration sense in the lower limbs), and significant weakness of bulbar muscles. According to the above criteria, it could be concluded that this patient's findings were consistent with those of MMNCB.

While the acute motor axonal (AMAN) variant of Guillain-Barre syndrome (GBS) is a differential diagnosis, the progression of symptoms without a plateau phase argues against AMAN. In addition, a decrease in CMAP amplitudes with relatively preserved motor latencies and motor conduction velocities is the electrophysiologic hallmark of AMAN. Chronic inflammatory demyelinating polyneuropathy (CIDP) may exhibit a conduction block since a conduction block is not exclusive to MMNCB. However, the sequential and asymmetric weakness in the index patient, with relative preservation of the distal latency and minor CSF protein elevation, suggests a diagnosis other than CIDP. Motor neuron disease (MND) is a differential diagnosis of MMNCB. Amyotrophic lateral sclerosis (ALS) and other lower motor neuron variants of MND, such as progressive muscular atrophy (SMA) and late-onset spinal muscular atrophy (SMA), are close differential diagnoses in patients with predominantly lower motor neuron signs [2]. Indeed, HIV-infected patients may develop motor neuron disease (MND) [15]. The absence of muscle atrophy and widespread fasciculations, with electrodiagnostic findings of multiple conduction blocks, points away from MND. Although IgM antibodies to ganglioside GM1, a supporting criterion, were not found in this patient, the clinical-electrodiagnostic picture met the diagnostic criteria for MMNCB. Anti-GM1 antibody is only found in about 50% of patients [16].

Of interest in the index case is the predominant involvement of the lower limb compared to the more usual upper limb presentation in typical cases. However, a third of patients could develop lower limb onset [5, 6]. While the first symptom of weakness is frequently seen in distal upper limbs with relative sparing of finger flexors, in those with lower limb presentation, foot drop is the first symptom, and they later develop upper limb symptoms, just like in the index case [16].

MMNCB presenting in a rare association with an NHL deserves extra attention, given that either condition would naturally call for a different treatment modality. Immunotherapy with IVIG is the mainstay of treatment for MMN, with satisfactory responses reported in up to 80% of patients [17-19], and chemotherapeutic agents are required in NHL; fortunately, the treatment of the NHL significantly improved this patient's neurological morbidity. Two chemotherapeutic agents in the R-CHOP regimen, rituximab and cyclophosphamide, have been tried with mixed results in the treatment of MMNCB. The apparent response to this regimen in our patient supports the efficacy of these medications in some circumstances. While rituximab and cyclophosphamide have been advocated for maintenance therapy of MMN in typical cases [20], they may be considered for induction and maintenance of therapy in malignancy-related cases. Whether the coexistence of the two conditions (NHL and MMN) was coincidental or the MMN was an epiphenomenon, we can establish that the chemotherapeutic agents alleviated both conditions.

Nevertheless, a confounding factor in our case was a new diagnosis of HIV. The influence of this diagnosis on the development, manifestation, and therapeutic response is poorly understood. To the best of our knowledge, MMN has not been characterized in the setting of HIV. Albeit, the peripheral nervous system is commonly involved in HIV [21]. Early on in the disease, focal neuropathy, brachial plexitis, and polyneuropathy, including GBS-like presentations [22], due to aberrant immune activation and inflammation during HIV seroconversion have been well-described [23]. Other patterns of involvement, such as subacute and chronic demyelinating polyneuropathy, distal symmetrical polyneuropathy, CMV polyradiculopathy, and late mononeuropathy multiplex, could occur later in the course of the illness [21, 23]. While polyneuropathy due to ART, especially the nucleoside analogs, is not uncommon, our patient did not receive any ART before her presentation, as she tested positive during her work-up of the index illness. Besides, ART-induced polyneuropathy is predominantly a sensory axonal polyneuropathy.

## Conclusions

Although a rare association, MMN could be a harbinger of lymphoma, as in this case. Further studies are needed to establish the relationship between NHL and MMN and how this could influence the treatment of either condition or their independent prognosis. We advocate for early evaluation of similar presentations and encourage reporting of such occurrences to enhance our understanding of this phenomenon.
